# A comprehensive monocentric ophthalmic study with Gaucher disease type 3 patients: vitreoretinal lesions, retinal atrophy and characterization of abnormal saccades

**DOI:** 10.1186/s13023-019-1244-9

**Published:** 2019-11-14

**Authors:** Susanne Hopf, Norbert Pfeiffer, Matthias Liesenfeld, Karl-Eugen Mengel, Julia B. Hennermann, Irene Schmidtmann, Susanne Pitz

**Affiliations:** 1 Department of Ophthalmology, University Medical Center Mainz, Langenbeckstr.1, 55131 Mainz, Germany; 2 Clinic for Anaesthesia, Surgical Intensive Care, Emergency Medicine and Pain Therapy, Klinikum Frankfurt Höchst, Frankfurt, Germany; 3 Villa Metabolica, Center for Pediatric and Adolescent Medicine, University Medical Center Mainz, Mainz, Germany; 4 Institute of Medical Biostatistics, Epidemiology and Informatics (IMBEI), University Medical Center Mainz, Mainz, Germany; 5 Orbital Center, Ophthalmic Clinic, Bürgerhospital Frankfurt, Frankfurt, Germany

**Keywords:** Saccadometry, Video-oculography, Saccades, Gaucher’s disease, Gaucher disease type III, Retina, Vitreous opacity, Ophthalmology, Neuro-ophthalmology, Lysosomal storage disease

## Abstract

**Background:**

The differentiation between Gaucher disease type 3 (GD3) and type 1 is challenging because pathognomonic neurologic symptoms may be subtle and develop at late stages. The ophthalmologist plays a crucial role in identifying the typical impairment of horizontal saccadic eye movements, followed by vertical ones. Little is known about further ocular involvement. The aim of this monocentric cohort study is to comprehensively describe the ophthalmological features of Gaucher disease type 3. We suggest recommendations for a set of useful ophthalmologic investigations for diagnosis and follow up and for saccadometry parameters enabling a correlation to disease severity.

**Methods:**

Sixteen patients with biochemically and genetically diagnosed GD3 completed ophthalmologic examination including optical coherence tomography (OCT), clinical oculomotor assessment and saccadometry by infrared based video-oculography. Saccadic peak velocity, gain and latency were compared to 100 healthy controls, using parametric tests. Correlations between saccadic assessment and clinical parameters were calculated.

**Results:**

Peripapillary subretinal drusen-like deposits with retinal atrophy (2/16), preretinal opacities of the vitreous (4/16) and increased retinal vessel tortuosity (3/16) were found. Oculomotor pathology with clinically slowed saccades was more frequent horizontally (15/16) than vertically (12/16). Saccadometry revealed slowed peak velocity compared to 100 controls (most evident horizontally and downwards). Saccades were delayed and hypometric. Best correlating with SARA (scale for the assessment and rating of ataxia), disease duration, mSST (modified Severity Scoring Tool) and reduced IQ was peak velocity (both up- and downwards). Motility restriction occurred in 8/16 patients affecting horizontal eye movements, while vertical motility restriction was seen less frequently. Impaired abduction presented with esophoria or esotropia, the latter in combination with reduced stereopsis.

**Conclusions:**

Vitreoretinal lesions may occur in 25% of Gaucher type 3 patients, while we additionally observed subretinal lesions with retinal atrophy in advanced disease stages. Vertical saccadic peak velocity seems the most promising “biomarker” for neuropathic manifestation for future longitudinal studies, as it correlates best with other neurologic symptoms. Apart from the well documented abduction deficit in Gaucher type 3 we were able to demonstrate motility impairment in all directions of gaze.

## Background

### Context and summary of the literature

Gaucher disease is the most common sphingolipidosis and is defined by the autosomal recessively inherited deficiency of lysosomal acid β-glucocerebrosidase. The gold standard for diagnosing Gaucher disease is the confirmation of deficient enzyme activity. Mutation detection in the affected GBA-gene can enhance the diagnostic accuracy [1]. However, there is a strong genetic heterogeneity of patients with similar symptoms and vice versa [2, 3]. Few genotypes are known to have a prognostic impact. The homozygote L444P allele is associated with Gaucher disease type 3 (GD3), which is the rare chronic neuronopathic form [4–7]. The diagnosis of Gaucher disease type 3 (GD3) is challenging as specific neurologic symptoms develop at various ages [8]. Reclassification of type 1 (visceral) to type 3 (chronic neuronopathic) in later stages occurs [9]. The most common early neurologic symptoms are oculomotor disturbances [10], such as slowed and hypometric saccades [8, 10] and saccadic initiation errors [8], and esotropia [8, 11] and/or abduction deficits [8, 12–14]. Horizontal saccades decelerate earlier, while vertical saccades follow [15–17]. Vertical downwards saccades are known to be impaired earlier than the less affected upwards saccades [8, 15, 16, 18]. As the clinical examination of oculomotor abnormalities has a limited reproducibility, depending on patient’s cooperation as well as the investigator’s evaluation, an objective oculomotor testing has the potential to result in a more objective assessment and a better quality of data for follow-up investigation [8].

Beside neuro-ophthalmologic abnormalities, Gaucher type 3 may present posterior segment abnormalities of the eye. Specific vitreous opacities are better known from Gaucher type 1, with a prevalence of 3% [19]. The prevalence of posterior segment abnormalities in Gaucher type 3 is not yet established. Recent case reports describe preretinal accumulations of matter [20–24]. In fact, they even may promote severe tractive retinal detachment in Gaucher type 3 [25, 26].

### Purpose of this study

The purpose of this study is to propose recommendations for a set of useful ophthalmologic investigations for diagnosis and follow up as well as saccadometry parameters of Gaucher disease type 3 patients enabling a correlation to disease severity.

## Methods

This monocentric cohort study was conducted in 2015 in the University Medical Center Mainz in Germany.

The study was approved by the Medical Ethical Committee of the State Chamber of Medicine of Rhineland Palatinate in Mainz, Germany (reference number 837.374.14 (9613)).

The patients or their parents/guardians gave a written consent to publication of their anonymized clinical data.

### Characteristics of participants

Inclusion criteria for participants were a biochemically and genetically diagnosed Gaucher disease type 3 and the cognitive ability to perform the investigations. Children under 6 years were excluded, because cooperation was regarded not to be sufficient for the examinations at that age. A visual acuity of better than 1.3 logMAR (at far distance) was required.

Sixteen patients were included in this study. A cohort of 100 healthy individuals at a stratified age distribution served as control.

### Ophthalmologic and neuro-ophthalmologic examination procedure

The patients underwent an ophthalmologic and neuro-ophthalmologic examination according to a standardized investigation form. Ophthalmologic examination included: refraction, best corrected visual acuity, anterior and posterior segment examination, Spectral Domain (SD)-OCT (Spectralis OCT, Heidelberg Engineering GmbH, Heidelberg, Germany) of the optic nerve head and the macula. Only OCT scans with a quality index above 20 were analyzed. Neuro-ophthalmologic examination included: orthoptic status (stereopsis, cover test, motility, optokinetic nystagmus and clinical saccade testing). A technical saccadometry was performed using an infrared-videooculography device (see below). Saccadometry was done by a specifically trained investigator. Orthoptic examination was carried out by an experienced orthoptist in 12 cases.

### Quantification of neuropathic Gaucher symptoms

The neuropathic Gaucher symptoms were assessed quantitatively by the disease specific “modified severity scoring tool” (mSST) using 12 categories [27]. Additionally, the “scale for the assessment and rating of ataxia” (SARA) including 8 items was assessed. Herein, the items ataxia and dysarthria [28] are characteristic in GD3 [29–31] and are also part of the mSST [27]. The IQ, one of the categories of the mSST, represents the cognitive function and in Gaucher patients serves to evaluate the neurologic involvement of the disease. The wide range of cognitive impairment in Gaucher patients has been reported by several authors [11, 15, 32].

### Video-oculographic saccadometry using EyeSeeCam in Gaucher patients

Saccades were recorded using the infrared video-oculography (VOG) device EyeSeeCam HIT (Eyeseetec, Fürstenfeldbruck), sampling at 220 Hz (every 3.6 ms) which was linked to a MacBook Pro 13″ (OS X Version 10.9.5, Apple Inc.) equipped with the EyeSeeCam software (EyeSeeCam VOG HIT System Reversion r3429 and r3444, EyeSeeTec Fürstenfeldbruck, Germany). The study participants were seated on a height-adjustable chair, facing the center of a computer monitor (Dell Technologies Inc., 19″ screen BQR-1908FPb, 1280 × 1024 resolution, 60 Hz refresh rate, 300 CD/m^2^ display luminance, and 5 ms images building time) at a distance of 60 cm from the glabella to the monitor. The linear visual range was at 5°/15°/30° leftwards and rightwards and 5°/10°/20° upwards and downwards. Each target was a smiley icon. Prior to the measurement, a qualitative calibration was performed. Movements of the left eye were measured. To avoid alternating fixation in heterotropia, the right eye was covered in these cases.

Saccades were counted and calculated at a threshold of 100°/s. The beginning and ending of a saccade was determined at a threshold of 5°/s. Further criteria were an occurrence 0.5 s after stimulus at latest and an amplitude of more than 0.5 of the relevant stimulus. In case that standard calculation did not detect enough saccades due to pathologically decelerated saccades, a “special saccades calculation” with more sensible criteria was used (threshold of 5°/s, beginning and end at 2°/s, latest detection 0.7 s after stimulus and detection of saccades even if they measure less than 0.5 of the stimulus amplitude).

The evaluation by “special saccades calculation” was necessary in 13/14 patients horizontally and in 8/14 patients horizontally to allow the detection of a sufficient number of saccades. Thereby a minimum of 4 saccades could be included in 82.4% of the 4 direction and 3 degree (12 different eccentricities) in the 14 patients.

### Statistical analysis, power calculation

With 16 patients and 100 controls, it was possible to establish a difference of 0.67 standard deviation between groups at 5% level with a power of 80% using a two sample t-test. This required that the parameter of interest followed a normal distribution with common variance. This study did not need an age matching as the main measurement parameters have been shown not to be age dependent in the targeted population (as described in a parallel paper) [33].

Statistical Package for Social Sciences (SPSS 23) was used for the analysis. To evaluate the distribution of data, Shapiro-Wilk-test and diagnostic plots were performed for each parameter to assess symmetry and peaks. Between the patient and the study group, normally distributed parameters were compared using t-tests. Within the patient group paired t-test was used. As this is an explorative study, we consider *p*-values ≤0.05 as indication for statistically significant difference. No adjustment for multiple testing has been applied, and thus only the error rate per comparison is controlled.

To assess correlations between the saccade parameters (peak velocity, amplitude, latency) and the mSST, SARA and IQ, Spearman correlation was performed. Spearman’s rank correlation coefficient of ≥0.8 was considered a strong correlation; ≥ 0.5 was considered a moderate correlation; ≥ 0.2 a weak correlation; and < 0.2 no correlation.

## Results

A total of 16 patients with biochemically and genetically diagnosed Gaucher disease type 3 was examined, of which 14 performed sufficient quality data in saccadometry. An ophthalmologic summary of the patient series is shown in Table 1, while Table 2 contains a detailed description of all patients.

**Table 1:** Ophthalmologic findings of all patients (*n* = 16)

Variable	Findings	Values/numbers
Demographics	Sex Age	9 female, 7 male (total: 16 patients) median 20.5 years (range 6 to 45 years).
Refraction	5 x myopic (OD + OS) 6 x emmetropic (OD + OS) 5 x hyperopic (OD + OS)	OD: median + 0.063D (range − 5.625D to + 2.125D), OS: median − 0.063D (range − 5.750D to + 3.125D). Gaucher vs. Control group (Wilcoxon test) *p* = 0.548 (OD) and *p* = 0.680 (OS)
Visual Acuity	normal	Median 0 OD: mean − 0.00625 ± 0.044, OS: mean 0.00625 ± 0.057
Anterior segments	Corneal opacity + slight retraction of the eye lids	1
Posterior segments	Vitreous opacities Epiretinal particles Retinal deposits+atrophy	4/16 3/16 ∑7/16 2/16
Oculomotor results	Abduction deficits Up−/Downgaze deficits	8/16 6/16
Saccadometry	Horizontal saccades Vertical saccades	15/16 abnormal 12/16 abnormal

**Table 2:** Clinical data of Gaucher type 3 patients

ID	Phenotype severity	Genotype	Age	Disease duration	SARA	IQ	Adjusted mSST	Motility (smooth pursuit)	Cover Test	Ocular findings	Horizontal verlocity (e.g. 30° rightwards)	Vertical velocity (e.g. 20° upwards)	logMAR visual acuity (right eye)	logMAR visual acuity (left eye)
1	Mild	L444P/ D409H	8	7	0	100	0	Full, synkinetic blinking, head thrust	Normal	Normal	22	337	0	0
2	Mild	L444P/ D409H	21	12	0	100	0.5	Full, synkinetic blinking, head thrust	Normal	Epiretinal particles on the ILM and posterior hyaloid, partially detached - > normal	46	293	0	0
3	Mild	L444P/ D409H	22	15	2	85	0.5	Full	Normal	Normal	57	266	0	0
4	Mild	N188S/L385P	24	3	0	100	1.5	Full	Normal	Partial vitreous detachment- > normal	332	323	0	0
5	Mild	–	27	21	0	100	0	OD: minimal adduction deficit, OS: minimal abduction deficit. OU: elevation 25°, depression normal. Synkinetic blinking.	Decompensating esophoria	OD: partial vitreous detachment, slight peripapillary atrophy, OD/OS: Lamina cribrosa- > normal	114	242	0	0
6	Mild	G202R/ D409H (*1)	44	38	39	71	18	*2: abduction 10°, adduction 20°, elevation 20°, depression 10°	Decompensating esophoria	Slight retraction of the eyelids, diffuse corneal opacity, tilted disc, prominent posterior hyaloid membrane, OD: large optic nerve head and excavation	25	39	0	0
7	Intermediate	L444P/ L444P	6	5	1	100	1	Full	Esotropia OD	Tortuous vessels	119	405	−0.1	−0.1
8	Intermediate	L444P/ L444P	7	6	0	104	1	Full	Normal	Tortuous vessels	119	452	−0.1	−0.1
9	Intermediate	L444P/ L444P	8	6	0	79	1	Full	Normal	Normal	88	333	0	0
10	Intermediate	L444P/ L444P	10	10	16	60	10	OD/OS: abduction 5° before primary position (p.p.)/p.p; adduction 30°/25°, minimal elevation and depression deficit	Esotropia OD (*4)	Normal (cave: bad image quality in OCT)	–	–	0	0
11	Intermediate	L444P/ L444P	12	12	10	68	6	Adduction 35–40°	Alternating esotropia	Large vitreous opacities, drusen-like deposits, retinal atrophy, partial vitreous detachment	98	134	0	0
12	Intermediate	L444P/ L444P	19	18	18	60	15	Full, synkinetic blinking	Esotropia OD (*4)	Large vitreous opacities	–	–	0.1	0.1
13	Intermediate	L444P/ L444P	21	19	4	75	5.5	Abduction 45°abduction 40°, adduction 35°, elevation 15° (*3), depression normal. Synkinetic blinking.	Alternating esotropia (*5)	Large vitreous opacities, tortuous vessels, epiretinal particles on an irregular ILM and the posterior hyaloid membrane	76	233	0	0
14	Intermediate	L444P/ L444P	23	23	0	100	2,5		Normal	Peripapillary atrophy due to high myopia - > normal	95	396	0	0
15	Intermediate	L444P/ L444P	33	31	9	68	18,5		Normal	Partial vitreous detachment, caliper changes, fine granular background, thin retina and thin vessels	91	173	0	0.1
16	Severe	–	19	18	7	79	12		Secondary alternating esotropia (*6)	Vitreous opacities, druse-like deposits, retinal atrophy (RPE and photoreceptor loss), enlarged optic nerve diameter (5.5 mm)	107	105	0	0.1

*1: G202R mutation known from GD2 with slightly reduced enzyme activity, while neuronopathic involvement is severe [34], *2: only elicitable by VOR, *3: elevation by VOR 25°, *4: patient had undergone once strabismus surgery OD, *5: patient had undergone 4 times strabismus surgery, *6: initially esotropia, Patients with ID 4, 6, 15, 16 were under anticonvulsive medication

**Fig. 1:**
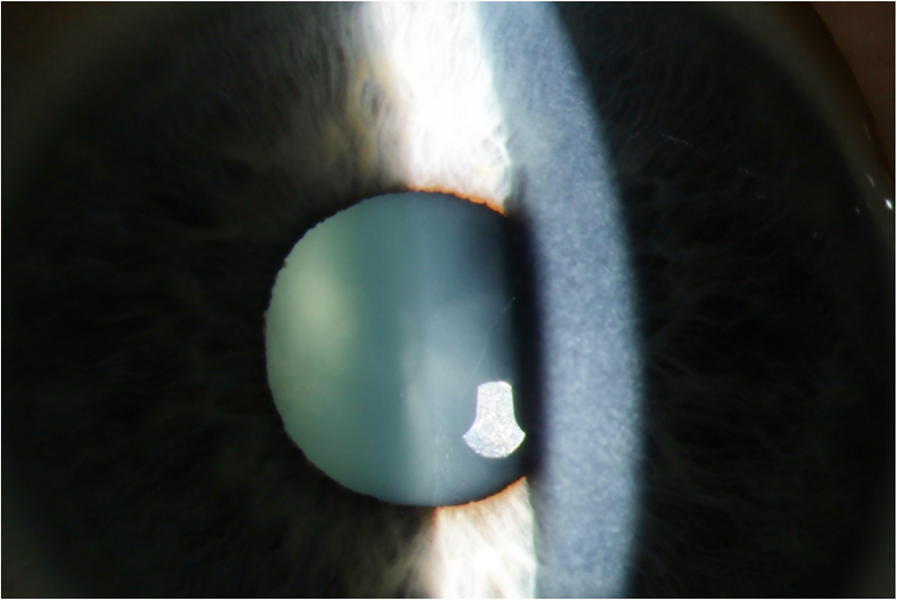
Diffuse corneal opacity in a Gaucher type 3 patient

In the anterior segment, a diffuse corneal opacity was found in one patient (Fig. 1) besides a slight retraction of the eye lids. The patient was heterozygous G202R/D409H. The corneal haze did not affect visual acuity (0 logMAR) nor did it affect OCT quality (quality index: 28/32 in the right/left macular OCT).

**Fig. 2:**
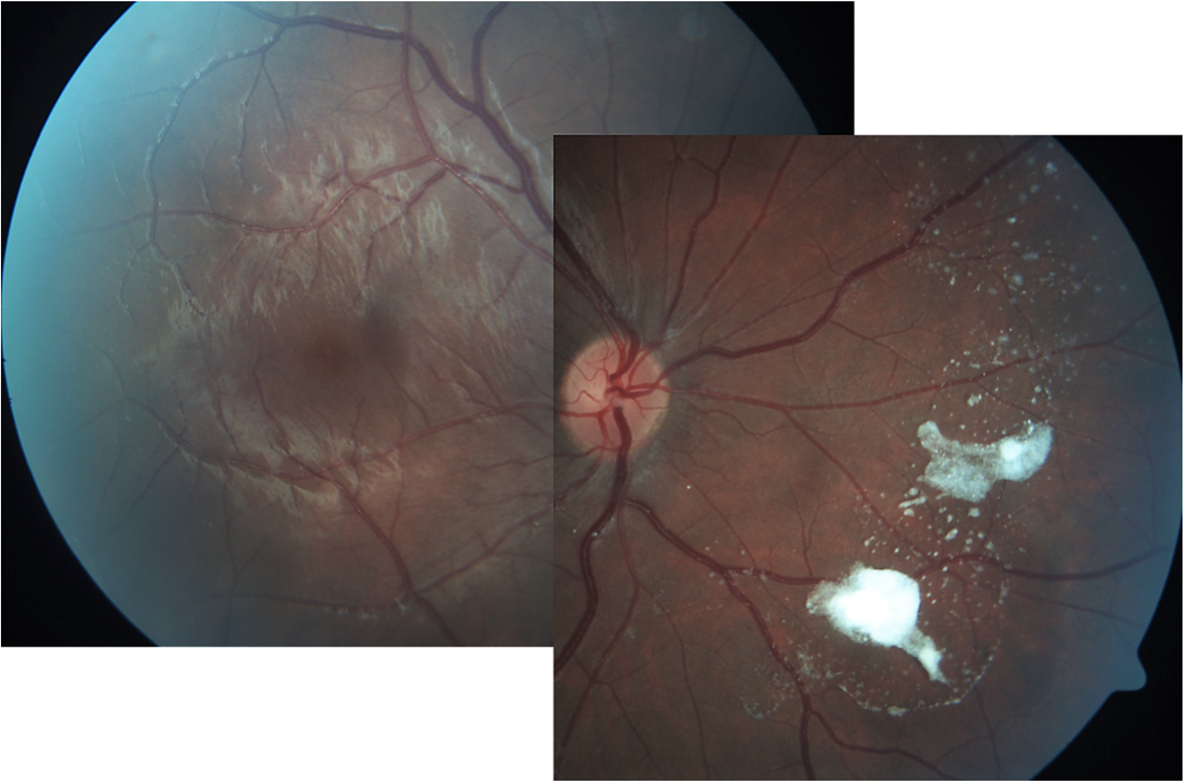
Vitreous opacity in a Gaucher type 3 patient

In the posterior segment, vitreous lesions were seen by fundoscopy or OCT. These mostly large opacities stood out as white or hyperreflective, often round, dot or cloud like opacities without disturbing patient’s subjective vision or visual acuity (Fig. 2).

In another three cases we found epiretinal particles on the ILM and the posterior hyaloid membrane by OCT. These lesions escaped fundoscopy.

**Fig. 3:**
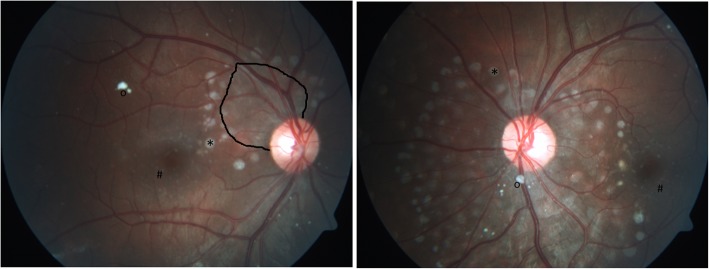
Posterior segment abnormalities. Drusen-like deposits (*) and retinal atrophy (demarcated area) in the peripapillary area between 9 and 1 o’clock. Small deposits in the perifoveal region (#). Vitreous opacity (o)

**Fig. 4:**
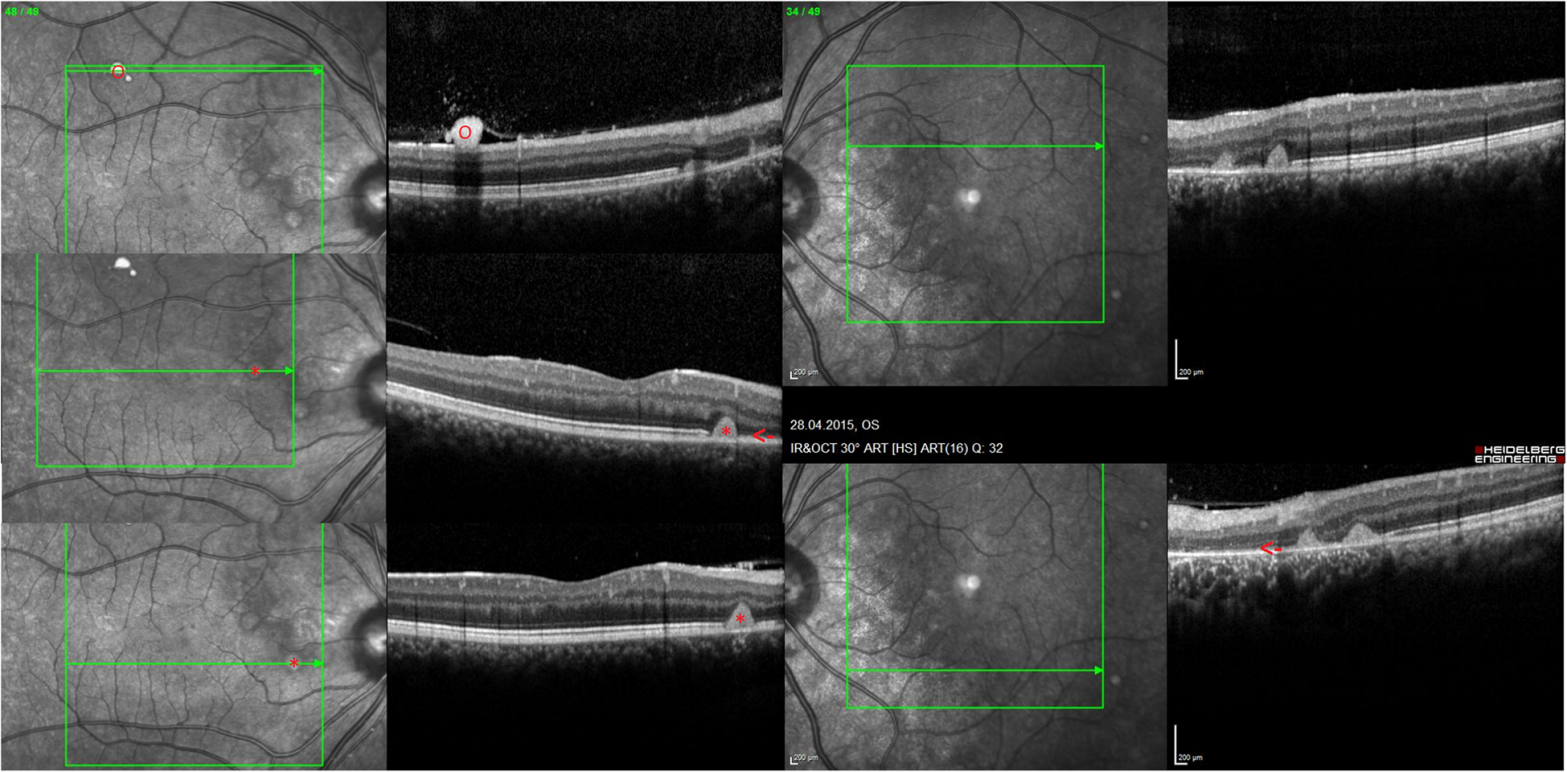
OCT of posterior segment abnormalities.Optical coherence tomography of the right and left eye showing subretinal dome shaped deposits (*) and adjacent retinal atrophy of the photoreceptor layer and retinal pigment epithelium layer (←). Vitreous (retrohyaloidal) opacity (o). Quality indices: right eye 28/34/34, left eye 32/36

**Fig. 5:**
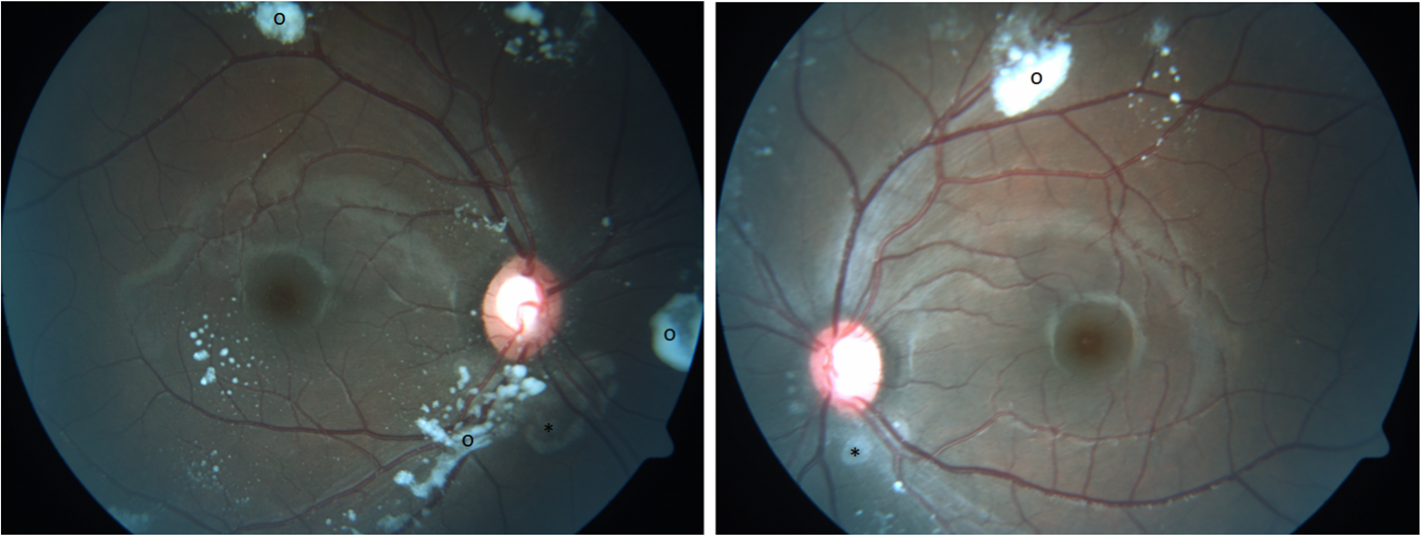
Posterior segment abnormalities in another Gaucher patient. Small subretinal deposits or atrophy (*) close to the optic nerve head and vitreous opacities (o)

**Fig. 6:**
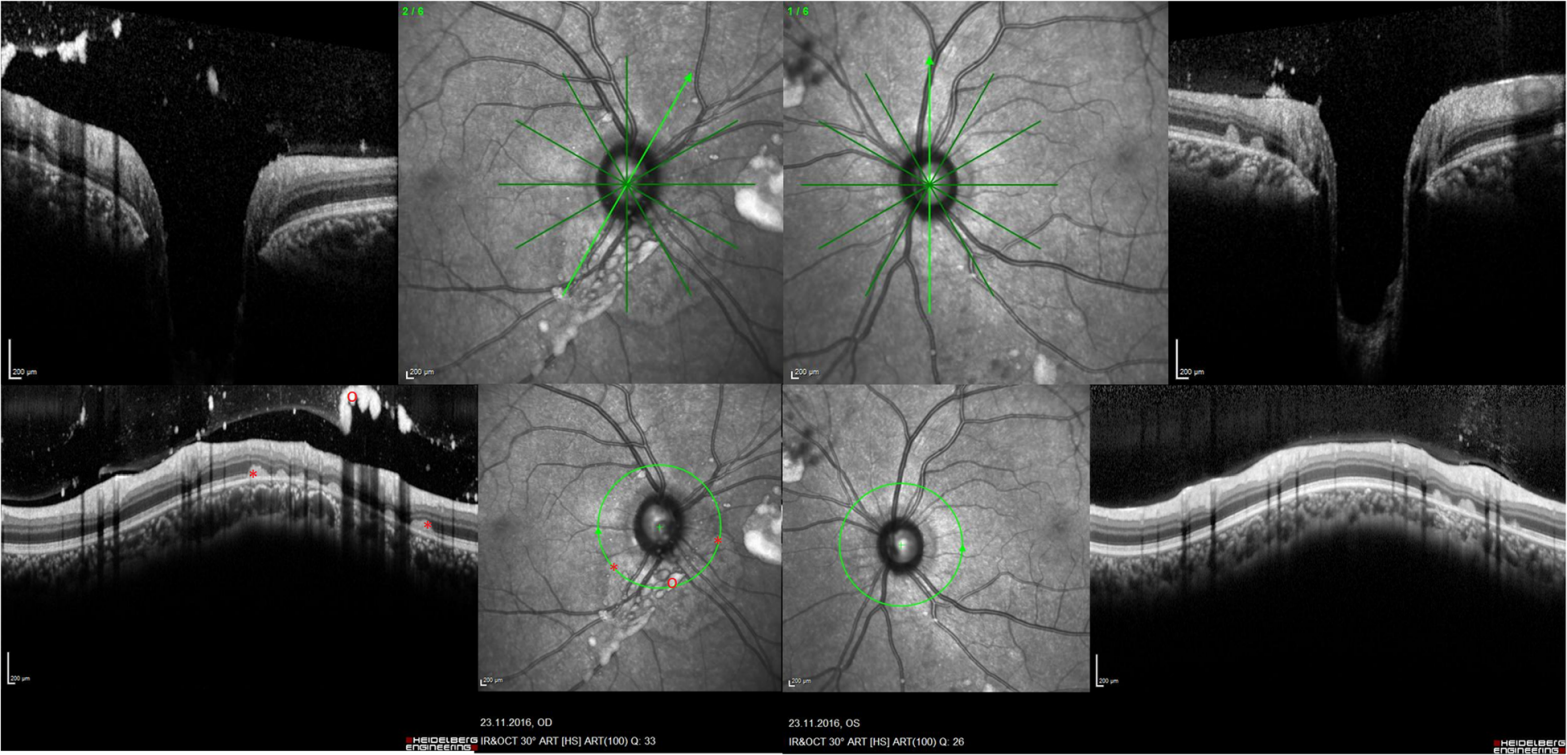
OCT of posterior segment abnormalities in another Gaucher patient. Optical coherence tomography of the right and left eye showing atrophy (←) and subretinal deposits (*) from 2 to 8 o’clock in the peripapillary area of the right eye. The left eye is less affected. Both eyes show remarkable vitreous opacities (o). Quality indices: right eye 31/33, left eye 32/26

While the previous ocular findings were located *preretinally*, two patients showed peculiarities, which were located *subretinally*. The lesions arose in the peripapillary region in a regular pattern in the shape of whitish drusen-like deposits, potentially reaching the macula area (Fig. 3). The deposits’ size was about 1/6 of the optic nerve head diameter which itself was slightly enlarged. Additional small lesions were found in the perifoveal area of the macula (Fig. 3). In the OCT, the deposits were identified in the subretinal region as hyperreflective dome shaped structures (Fig. 4). Importantly, the retina adjacent to these deposits was atrophic, showing photoreceptor and retinal pigment epithelium (RPE) loss in an extended area towards the optic nerve. The more affected case was a patient with a severe phenotype (Fig. 3), while the other one had intermediate phenotype and presented few deposits and retinal atrophy close to the optic nerve head in addition to extensive vitreous opacities (Figs.5 and 6). Both patients had a comparable disease duration of about 19 years.

Tortuosity or caliper changes of retinal vessels were detected in four patients (Additional file 1).

Optic nerve head appearance was normal; two patients exhibited tilted discs due to moderate/high myopia. All optic discs had normal retinal nerve fiber layer (RNFL) thickness in OCT.

### Oculomotor investigation

Bilateral abduction deficits were found in 8/16 patients, while 6 patients additionally presented downgaze or/and upgaze restriction. While patients with mild phenotype show free motility until the middle of the 3rd decade, restricted motility already occurs in the 2nd decade in patients with a severe phenotype (Table 2). The motility deficit was of marked severity in two of the patients precluding saccadometric measurements due to poor quality. Strabismus was present in 8 of 16 patients (most common strabismus convergens, followed by strabismus convergens alternans and decompensated esophoria). Half of the patients had full stereopsis in Titmus ring test, however stereo resolution was significantly worse than in the control group (mean degree of disparity in patients 130 (± 149) arc seconds vs. 54 (± 45) arc seconds in the controls, *p* = 0.041). Limited triggerable optokinetic nystagmus was seen in 14/16 patients (stronger impairment horizontally in 8/16).

Binocular clinical assessment revealed horizontal saccades to be more often decelerated (15/16) than vertical ones (12/16) (Table 3). The patient presenting normal saccades horizontally suffered epilepsy and was genetically diagnosed as type 3 as both mutations N188S and L395P are known in type 3 GD. No type 1 mutation/ no neuroprotective mutation was present.

**Table 3:** Clinical saccade testing of Gaucher patients

Clinical saccade testing	Horizontally (rightwards/ leftwards)	Vertically (upwards/ downwards)
(0) normal	1	4
(1) slowed slightly	1	7
(2) slowed a lot	9	upwards 3/ downwards 2
(3) not triggerable	3	upwards 2/ downwards 3
(4) not assessable	2	0
*Total*	*16*	*16*

Rightwards and leftwards saccades were comparable in all patients, however in the above mentioned two patients not assessable due to marked motility restriction. In three patients horizontal saccades were triggerable exclusively by the use of blinking. Two of them also presented head thrust.

### Saccadometric measurements

A minimum of 4 recorded saccades (as response to 6–8 stimuli per direction and target displacement) in each of the 16 patients was detected in 82.4% of the 12 eccentricities in 16 patients (98.5% in the 100 control subjects). Missing values were few. The saccade parameters had an approximately normal distribution except for latency.

**Fig. 7:**
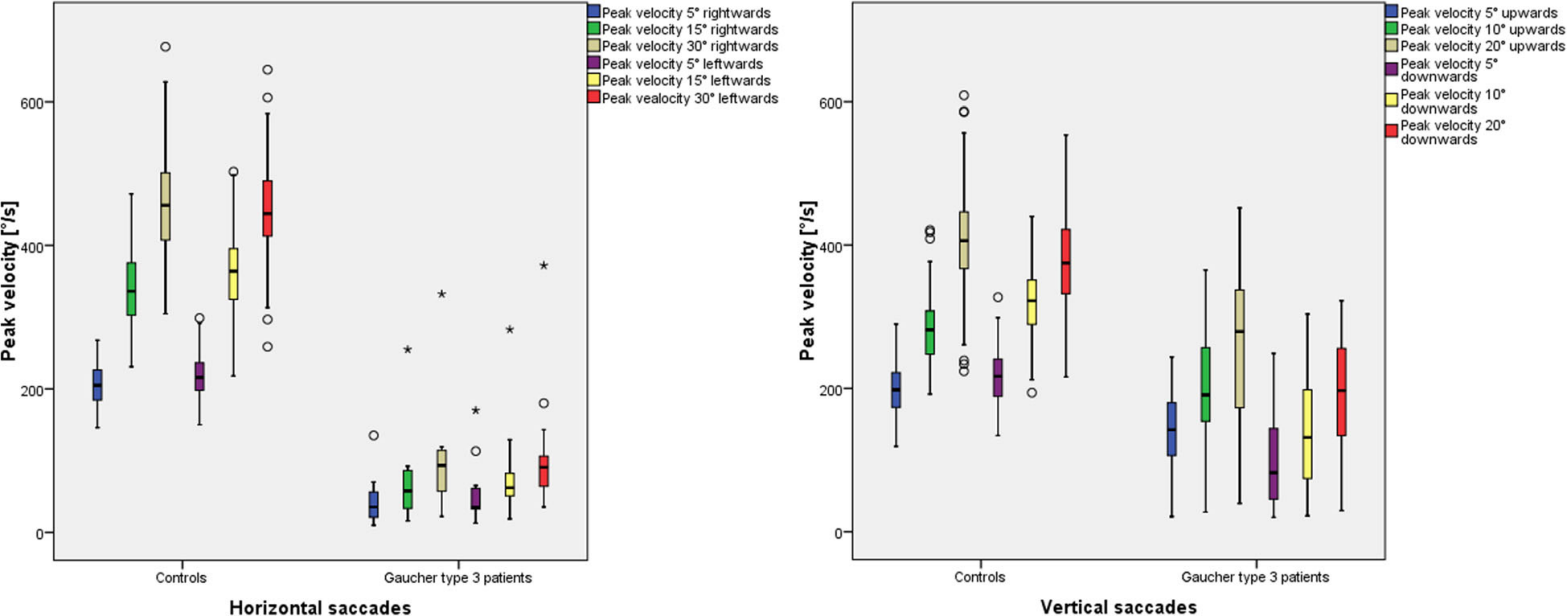
Saccadometric peak velocity in Gaucher type 3 patients compared to 100 controls split by target excentricity. The peak velocity (y-axis) is displayed for Gaucher type 3 patients vs. the control group for different target eccentricities. The whiskers extend to the minimum and maximum, when there are no outliers. Outliers (and extreme values) are displayed as circles (asterisks), meaning values defined by a distance of more than 1.5 times (3 times) the interquartile distance from the box (from the median). The lower boundary of the box is the 25th percentile (25% quartile), the line within the box indicates the 50th percentile (median) and the upper boundary represents the 75th percentile (75% quartile). The arithmetic mean is shown as asterisk. In all age groups, the peak velocity increases with increasing target eccentricity

Saccade parameters, especially peak velocity, showed to be impaired in a characteristic pattern of damage: horizontally (rightwards/leftwards) followed by downwards and last upwards (Fig. 7).

Peak velocity was significantly reduced (exemplarily peak velocity 15° saccades horizontally: 69°/s rightwards and 83°/s leftwards in GD3 patients vs. 339°/s rightwards and 364° leftwards in the controls, *p* ≤ 0.001), while main sequence was preserved. The difference to the control group was more evident horizontally and downwards (*p* ≤ 0.001), followed by upward saccades (*p* = 0.001–0.003). Moreover, paired t-test showed downwards saccades in GD3 patient to be slower (144°/s ± 87°/s) compared to upwards saccades (192°/s ± 94°/s).

Further saccadometric data are provided in the Additional file 2 displayed as boxplots [see Additional file 2]. Gain or saccadic accuracy as defined per amplitude divided by target eccentricity proved to have a larger range (in terms of interquartile distance) in GD3 patients. The saccades were of similar accuracy vertically, while being less accurate horizontally. Both study groups showed hypomentric saccades more often in horizontal than in vertical direction (see Additional file 2A).

The latency of Gaucher patients was not normally distributed. The non-parametric Wilcoxon rank sum test revealed that latency was significantly longer in Gaucher patients compared to controls except for upward direction and large target displacements (20° vertically and 30° horizontally) (Table 4) (see Additional file 2B).

**Table 4:** Latency of Gaucher patients vs. controls computing Wilcoxon rank sum test

Target eccentricity	5 ° R	15 ° R	30 ° R	5° L	15° L	30° L
P-value (t-test)	*0,013	*0,066	0,768	*0,004	*0,003	0,147
Target eccentricity	*5° UP*	*10° UP*	*20° UP*	*5° DOWN*	*10° DOWN*	*20° DOWN*
P-value (t-test)	0,128	0,584	0,855	*0,048	*0,071	0,817

### Correlation analysis between saccade parameters and other neurologic parameters

Clinical scores correlated with subjective and objective saccadic peak velocity and with saccade hypometria. Phenotype severity showed mild correlation with longer latency. High mSST (adjusted for saccades), high SARA, long duration of disease and low IQ correlate with slow and hypometric saccades. The correlation was found to be greater in vertical than in horizontal saccades. Phenotype severity correlated with longer latency, while its correlation with other saccade parameters was not universal. The underlying analyses data are provided in the Additional file 3, and the neurologic items are listed in the Additional file 4 [see Additional files 3 and 4].

## Discussion

We report ocular involvement and saccadometric parameters in this to date largest monocentric ophthalmologic cohort of GD3.

### Ophthalmologist’s findings

Diffuse corneal opacities have been infrequently reported in GD 3 patients [24]. They occurred in D409H homozygous patients [35–38] in association with cardiac valve calcification, while we found corneal haze in a heterozygous patient as did Inui et al. in toddler Gaucher type 2 [39]. Our heterozygous patient with corneal opacity was significantly older (44 years) than other patients. If the corneal opacity is due to the presence of the D409H mutation (albeit in the heterozygous compound state), it could be hypothesized that the absence of this finding in the other carriers of the same mutation (L444P/D409H) might be due to their still young age. In another case of Gaucher type 1 disease (F216Y/L444P), corneal abnormality preceded the diagnosis almost 15 years [40].

First descriptions on posterior segment abnormalities in Gaucher patients, before enzyme replacement therapy was established, focused on vitreous opacities [41, 42]. Recent case reports confirmed vitreous opacities, condensations, preretinal hyperreflective dots and posterior vitreous detachment by OCT [21, 23] [24]. Sheck et al. [23] located a hyperreflective preretinal accumulation between the posterior hyaloid interface and the nerve fiber layer of the retina in the mid peripheral and perimacular area (as also shown in Fig. 3). An additional sign in this 14-year-old girl was partial posterior vitreous detachment [23]. One year later in 2013, Coussa showed comparable OCT recordings in a 13-year-old girl – *preretinal* deposits - with slight progression after a follow up period of 5 years [21]. These are in accordance with our experiences in 16 GD3 patients, in which we found epiretinal particles in the macula, epiretinal and vitreous opacities in the mid periphery, and partial detachment of the posterior vitreous. However, in the present study, we did not only see *vitreoretinal lesions* (at the interface*)*, but also *subretinal lesions*. Their occurrence seems more typical with severe phenotype and longer disease duration. The OCT characteristics seem similar to drusen but unlike drusen, the color was whitish instead of yeallowish, similar to the pre-retinal presumed Gaucher cell bodies. One could speculate that the drusen-like deposits are Gaucher cell collections. These lesions located at the peripapillary region, reaching the macula were accompanied by retinal atrophy per loss of photoreceptors and retinal pigment epithelium. We were unable to find comparable findings in a computerized literature search. While chorioretinal atrophy is described in other storage diseases [43], their appearance in these diseases is considerably less marked and without deposits.

Likewise, tortuosity of the retinal vessels is known from other lysosomal storage diseases such as Fabry disease and alpha-mannosidosis. Unlike the isolated finding of tortuosity in our cohort, a previously reported case of GD showed severe tortuosity with extensive opacities in the vitreous body accompanied by visual impairment [44]. Red cherry spot is no specific sign for Gaucher disease [24], which we could confirm. In our cohort, we could not detect any nerve fiber layer deposits as described by Sawicka-Gutaj et al. [22].

At the time being, we cannot answer the crucial question whether the occurence of subretinal (or vitreoretinal) affection indicates a neuronopathic (or visceral) manifestation.

Watanabe [25] and Zhao [26] described tractional retinal detachment due to strong vitreoretinal adhesions and massive vitreous opacities in GD3. In teenagers, the underlying mechanism might include early development of liquefied cavity that exercises traction by the vitreous [25]. If retinal detachment develops, surgical treatment will be essential to preserve vision [25], otherwise permanent vision loss might occur at a young age [26].

Therefore, from the ophthalmologist’s point of view, Gaucher patients whether they are diagnosed type 1 or 3, should undergo the following diagnostic procedures:

- Macula and papillary dense optical coherence tomography (the resolution of OCT is 7–25 μm, which makes it a sensitive technique) helps to localize subretinal and vitreoretinal lesions.
- Fundoscopy (with dilated pupils) and fundus pictures enable to document the extent of the lesions in selected cases for follow-up. Differential diagnosis of these lesions – especially the distinction from intraocular lymphoma – is challenging, as GD patients are known to have an increased risk of haematological malignancies [24]. Ideally, fundus photography should be performed by obtaining coloured and red-free recordings, as the case may be.
- Electroretinogram and visual field investigation might be informative to detect retinal involvement and optic disc anomalies or in case of otherwise unexplained visual impairment.

### Neuroophthalmologic aspects

GD3 patients develop a progressive horizontal supranuclear gaze palsy [15, 45]. Motility restriction, namely bilateral abduction deficits, indicate abducens motoneuron/nucleus affection [12], esotropia being the likely result of this N. abducens involvement. We found esotropia or decompensated esophoria in 8/18 patients (44%), which is in a comparable range as described earlier [11], and considerably more frequent than its prevalence in healthy subjects (2–3.5%). We could additionally demonstrate motility restriction involving all directions of gaze in 6/16 patients (38%). Oculomotor disturbances follow a typical pattern in Gaucher type 3, in accordance to the topo-anatomical areas (PPRF (paramedian pontine reticular formation), riMLF (rostral interstitial nuclei of the medial longitudinal fasciculus), motoneurons of the abducens nucleus, flocculus/cerebellum and vestibular system [12, 18]. Besides slowed saccadic velocity, the initiation of saccades is delayed and [10] and saccadic gain (accuracy) is reduced, horizontally more severely than vertically [15]. Horizontal gaze may be affected so severely that technical measurement is impossible. Therefore, being a more sensitive measurement, the investigation of the less impaired vertical saccades has gained much more attention in saccadometric studies or possible effects of medical treatment [15, 17]. 2/16 of our patients exhibited saccades that were not reliably measurable due to motility restriction.

### Saccade parameters: peak velocity, latency and gain

*Peak velocity:* The relation between increasing peak velocity by increasing amplitude, termed main sequence in healthy subjects [46], is preserved in GD3 patients despite abnormal saccades. The overall saccadometric results of decelerated horizontal and secondary vertical saccades confirms previous data on slowed saccades in GD3 patients [8, 10, 18].

*Latency*: As in previous reports, latency was found to be prolonged [10, 12, 15, 18], except for upwards saccades, which impair lastly. The results show enlarged interquartile distances in the boxplots, which in our view rather reflects the susceptibility for confounders such as age than for disease severity.

*Gain:* Saccadic accuracy (gain) received relatively little attention in studies on oculomotor involvement in GD3 patients. A study on Norbottnian GD3 has recently reported normal accuracy [18], while a previous report described it to be reduced [15]. In our cohort, horizontal saccades were less precise and rather hypometric. The same held true for vertical ones, although to a lesser extent. One of the reasons for low gain values in general is motility restriction, especially regarding the maximal target eccentricity. Anticonvulsive medication might be a confounder resulting in slowed and hypometric saccades [45]. We figured out the patients without anticonvulsive medication (12/16) to have slowed saccades (just like the patients with anticonvulsive medication (4/16)) with one exception corresponding to a mild genotype (N188/−).

### Saccadometry is useful

Saccadometry was feasible in all Gaucher type 3 patients (14/16 performed saccadometry with sufficient data quality). We also recommend performing OCT as it is a widely used, expeditious, and informative investigation method feasible in less than 10 min in Gaucher patients, while saccadometry rather takes 10 to 30 min depending on the protocol used and the necessity to retake a test. The additional, typical signs of oculomotor abnormalities such as synkinetic blinking [8] are assessable by clinical evaluation. Another technique is an upwards curvature to horizontal saccades likely a Bell’s phenomenon (but keeping the lids open). Both are felt to inhibit the omnipause region allowing the saccade to begin. The latter phenomenon is noticeable during saccadometry and may disturb measurements by producing artefacts. Clinical assessment is difficult and less sensitive: While clinical assessment could not reveal differences between upwards and downward saccades, saccadometry was able to measure significant differences regarding this measurement. This is why we recommend to screen Gaucher type 1 patients and to monitor Gaucher type 3 patients for their neurologic manifestation by meticulous motility evaluation, ideally by video-oculography. We suggest that GD1 patients should be assessed for individual follow-up and that a normative GD1 cohort data, which does not exist to the best of our knowledge, should be established.

Infrared video-oculography is non-invasive, not stressful and easy to understand. It is well tolerated by children, however case-by-case-decision is surely advocated. The measurements are time consuming and editing of the raw data is needed to ensure optimum data acquisition. The quality of measurements depends primarily on the patient’s cooperation of the investigator’s experience.

Correlation between peak velocity and neurologic status (adjusted SARA, disease duration, IQ and to a slightly less extend the mSST) was greatest vertically. We were unable to reproduce the strongest correlation for downwards saccades as reported by Bremova (2018) [12]. However, the better preserved vertical spectrum of velocity allows stronger correlations. This fact has been explained by the horizontal floor effect [15] or ceiling effect [12]. Vertical saccadic peak velocity therefore functions as indicator for neurologic manifestation [12, 15, 17]. Saccadometry has the potential to qualify for those diagnostic procedures allowing quantification of neurologic manifestation such as the mSST. It might replace the less objective clinical saccade assessment and optimize the scoring system. Although correlation between clinical assessment of saccadic impairment and adjusted mSST, SARA and IQ were demonstrated, saccadometry is definitely more objective.

### Strengths and limitations of this study

Despite the low prevalence of all GD-phenotypes of 0.7 to 1.75 per 100.000 inhabitants [47] and an amount of 5% GD3 [48] within these, we were able to investigate a group of 16 GD3 patients. Most studies reporting on oculomotor disorders in GD3 are based on smaller samples [8, 10, 14, 15, 18] or display a multicenter study design [12]. Schiffmann et al. performed saccadometry on 30 GD3 patients in two study centers as part of a randomized controlled study on miglustat [17]. One older report exists on clinical oculomotor and ophthalmological findings in 22 Norbottnian GD3 patients [11]. Statistical analysis of the limited samples encountered in rare diseases is challenging. In this explorative study, the significance level has not been adjusted for multiple testing, which may result in an excess of false positive results. Confirmatory studies are required to verify the results.

Another problem is a possible selection bias as the included patients mostly presented mild or intermediate phenotype severity. For patients with severely affected oculomotor disturbances, a favorable atmosphere for the measurement was created and the eyelid was fixed to minimize blinking artefacts.

A potential flaw of our investigation is the fact that in presbyopic study participants, no near correction was used. Quite interestingly, up to now there is no data on how near correction influences saccadometric precision [49]. Myopes and emmetropes show similar saccadic eye movements [50].

Regarding the pre-set program of the video oculography system we used, it would be useful to provide a shorter protocol to maintain attention and thus to reduce artefacts during saccadometry in patients with abnormal eye movements. As patients with restricted motility are not able to reach large target eccentricities, smaller maximal target displacements should be considered in those cases. Longitudinal saccadometry might be appropriate for monitoring and seems to be more sensitive than the clinical testing used in the scoring system.

## Conclusions

Characteristic ocular manifestations of Gaucher disease type 3 may present as vitreoretinal lesions (vitreous opacities) and subretinal lesions (deposits with retinal atrophy in advanced disease stage). We recommend an ophthalmologic assessment including fundoscopy with dilated pupils and optical coherence tomography (optic nerve head and macula). The pattern of saccadic impairment in Gaucher type 3 affects predominantly horizontal, later downwards and finally upwards eye movements. Peak velocity vertically as a biomarker for neuropathic manifestation is valuable for future longitudinal studies, as it correlates best with other neurologic symptoms. We could additionally demonstrate motility restriction to involve all gaze directions, not only abduction deficits which cause esotropia in Gaucher patients. This study is valuable for ophthalmologists because they can contribute to diagnosis of Gaucher type 3 by neuro-ophthalmologic examination and may alert in case ocular manifestations are present indicating severe disease stage.

## Supplementary information

**Additional file 1.** Retinal vessels in Gaucher type 3 patients (Figure).

**Additional file 2:** Saccadometric data including gain (A) and latency (B) in Gaucher type 3 patients compared to 100 controls split by target eccentricity (Boxplots).

**Additional file 3:** Correlation analysis between saccade parameters and other neurologic items (Table).

**Additional file 4:** Neurologic items in Gaucher type 3 patients (Table).

## Data Availability

Data generated or analyzed during this study are included in this published article.

## Abbreviations

GD3: Gaucher disease type 3
mSST: Modified severity scoring tool
mSST: Modified severity scoring tool
OCT: Optical coherence tomography
PPRF: Paramedian pontine reticular formation
riMLF: Rostral interstitial nuclei of the medial longitudinal fasciculus.
RNFL: Retinal nerve fiber layer
RPE: Retinal pigment epithelium
SARA: Scale for the assessment and rating of ataxia
