# Supplementary material for: A comprehensive monocentric ophthalmic study with Gaucher disease type 3 patients: vitreoretinal lesions, retinal atrophy and characterization of abnormal saccades
Source: Orphanet J Rare Dis. 2019 Nov 14;14:257. doi: 10.1186/s13023-019-1244-9 (PMC6857165; doi:10.1186/s13023-019-1244-9)
Supplement: Supplementary file 1 — Additional file 1. Retinal vessels in Gaucher type 3 patients (Figure). [file 13023_2019_1244_MOESM1_ESM.docx]

**Additional file 1: Retinal vessels in Gaucher type 3 patients.**

**C**


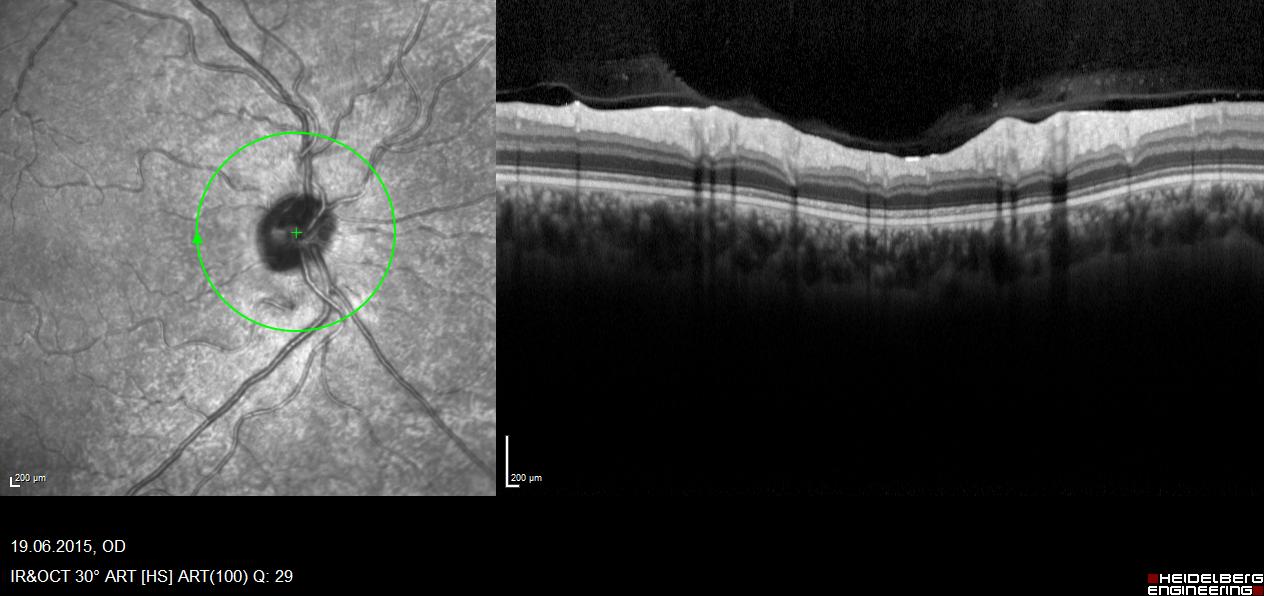


**B**

**A**


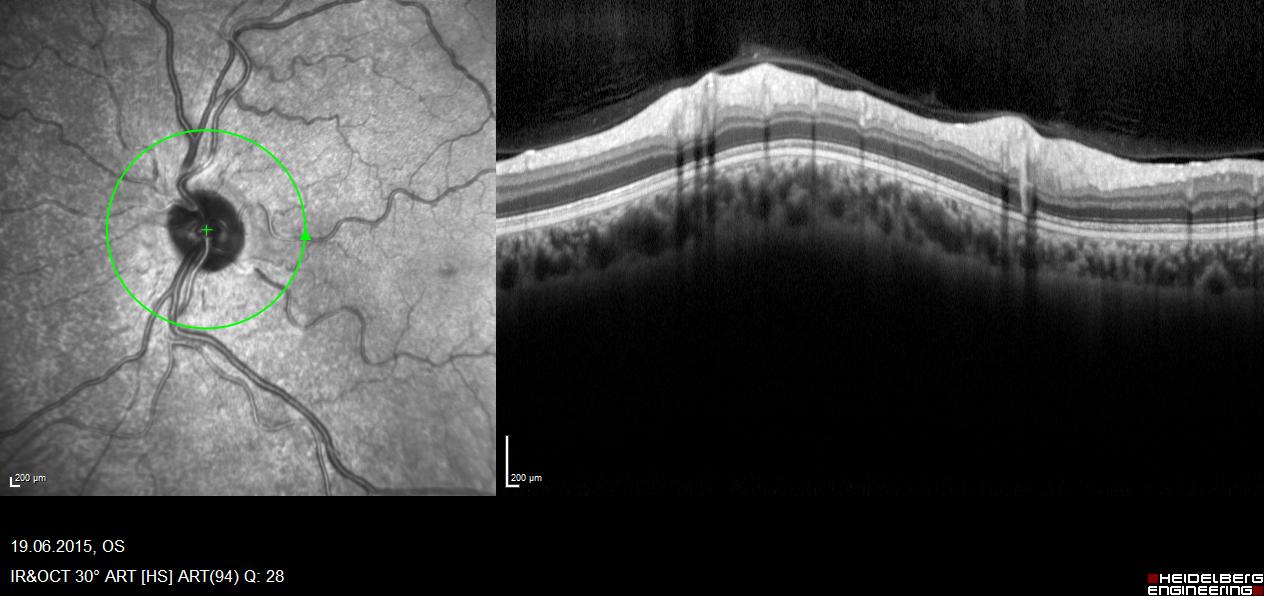

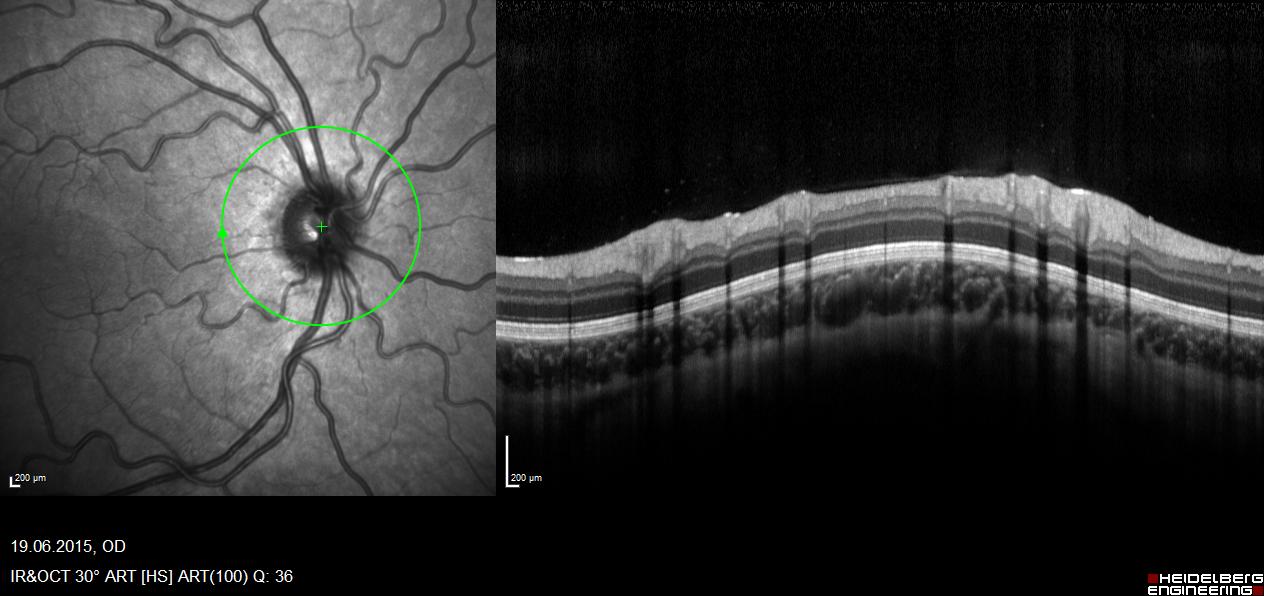

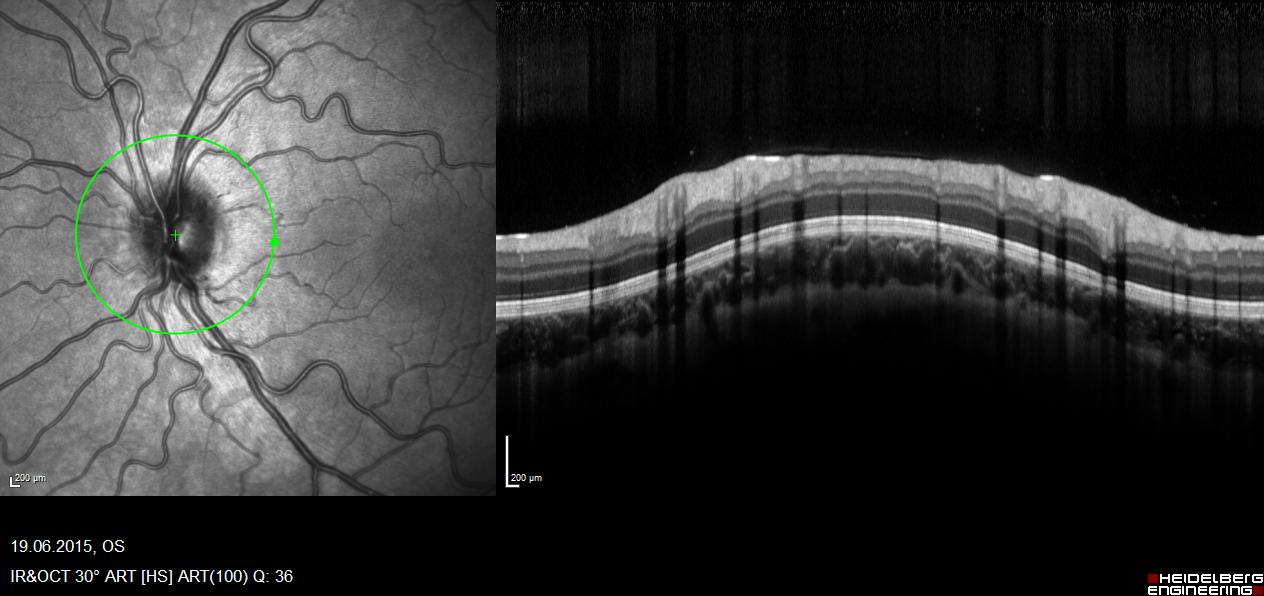

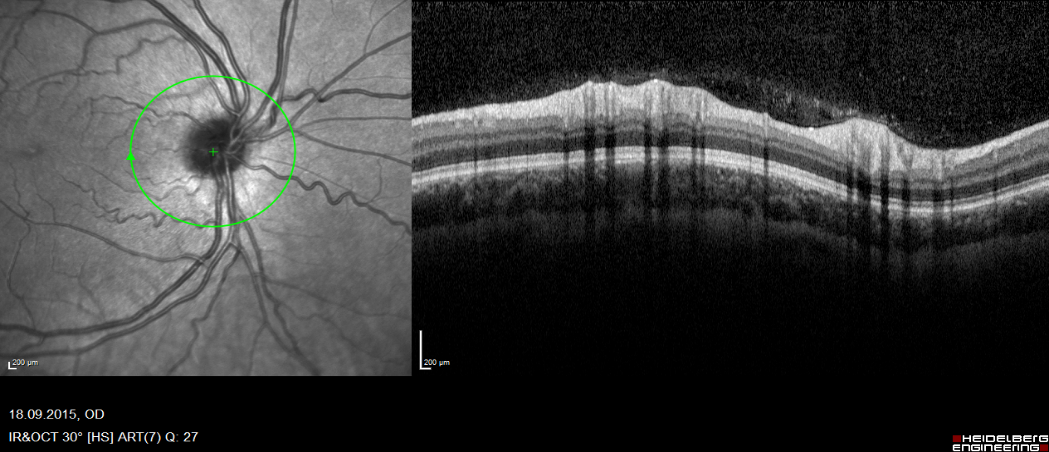

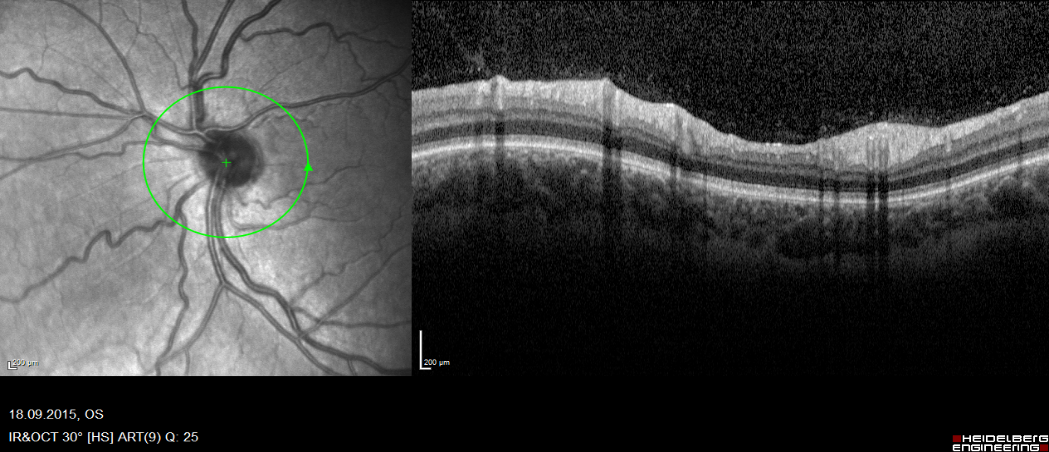


**D**


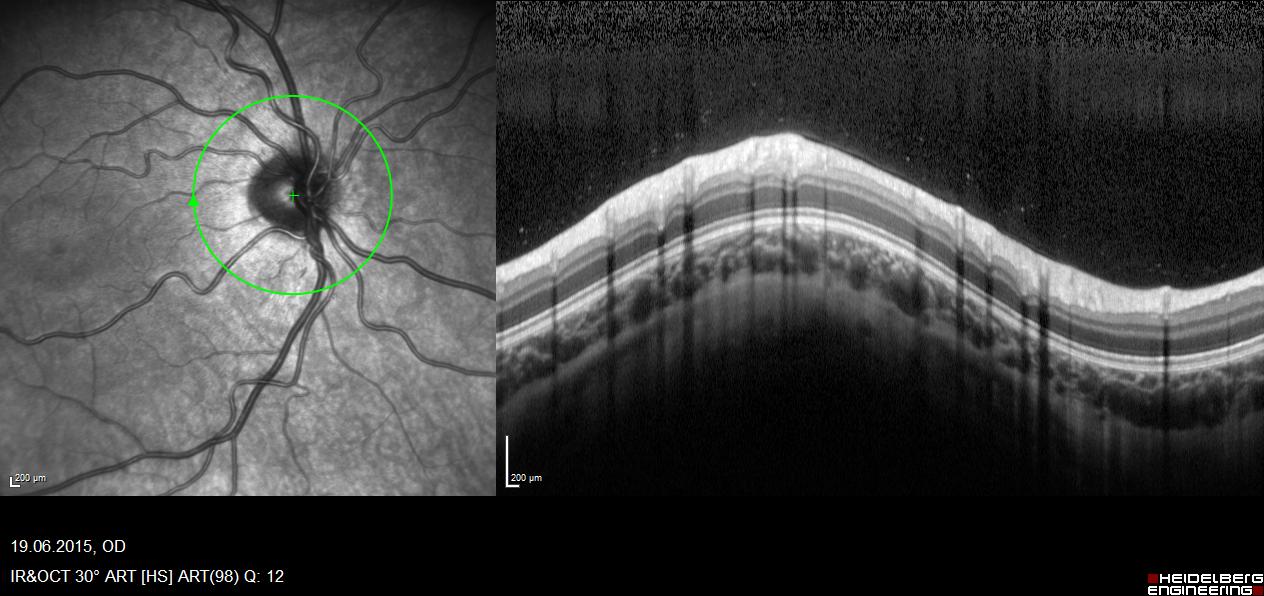

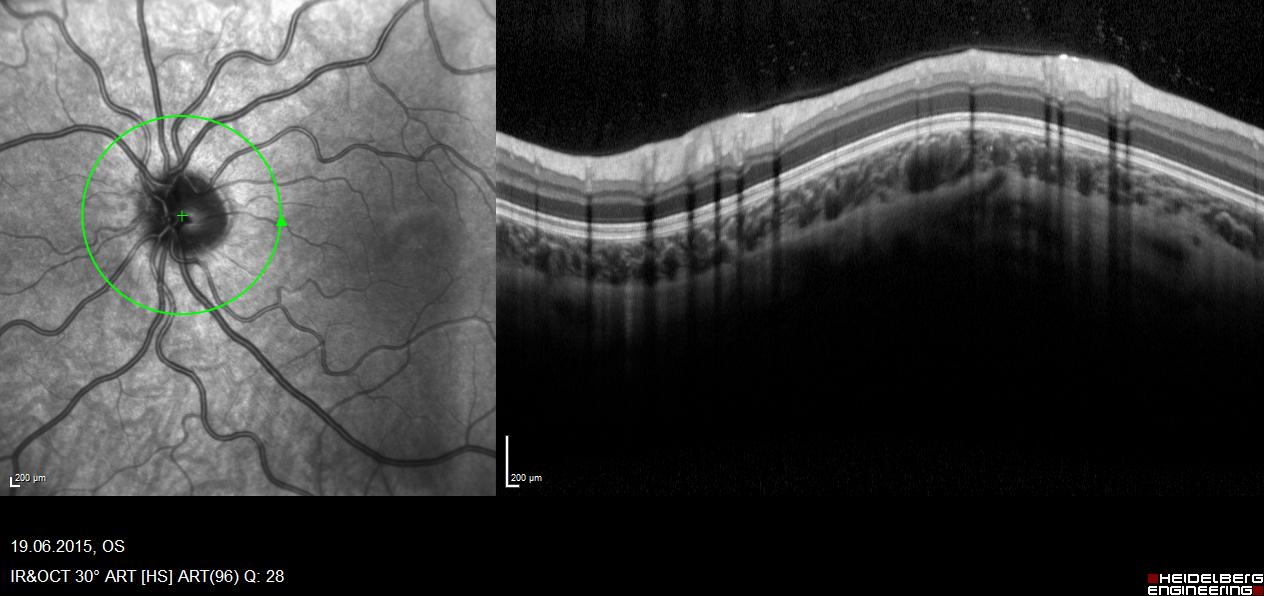


*Infrared images (recorded by OCT) focused on the optic nerve head with the central retinal vessels, showing caliper changes of retinal vessels on a fine granular background (A) (Patient No. 15) and tortuous vessels (B/C/D) (Patient No. 13/8/7).*
