# Supplementary material for: A comprehensive monocentric ophthalmic study with Gaucher disease type 3 patients: vitreoretinal lesions, retinal atrophy and characterization of abnormal saccades
Source: Orphanet J Rare Dis. 2019 Nov 14;14:257. doi: 10.1186/s13023-019-1244-9 (PMC6857165; doi:10.1186/s13023-019-1244-9)
Supplement: Supplementary file 2 — Additional file 2: Saccadometric data including gain (A) and latency (B) in Gaucher type 3 patients compared to 100 controls split by target eccentricity (Boxplots). [file 13023_2019_1244_MOESM2_ESM.docx]

**Additional file 2: Saccadometric data including gain (A) and latency (B) in Gaucher type 3 patients compared to 100 controls split by target eccentricity.**

**
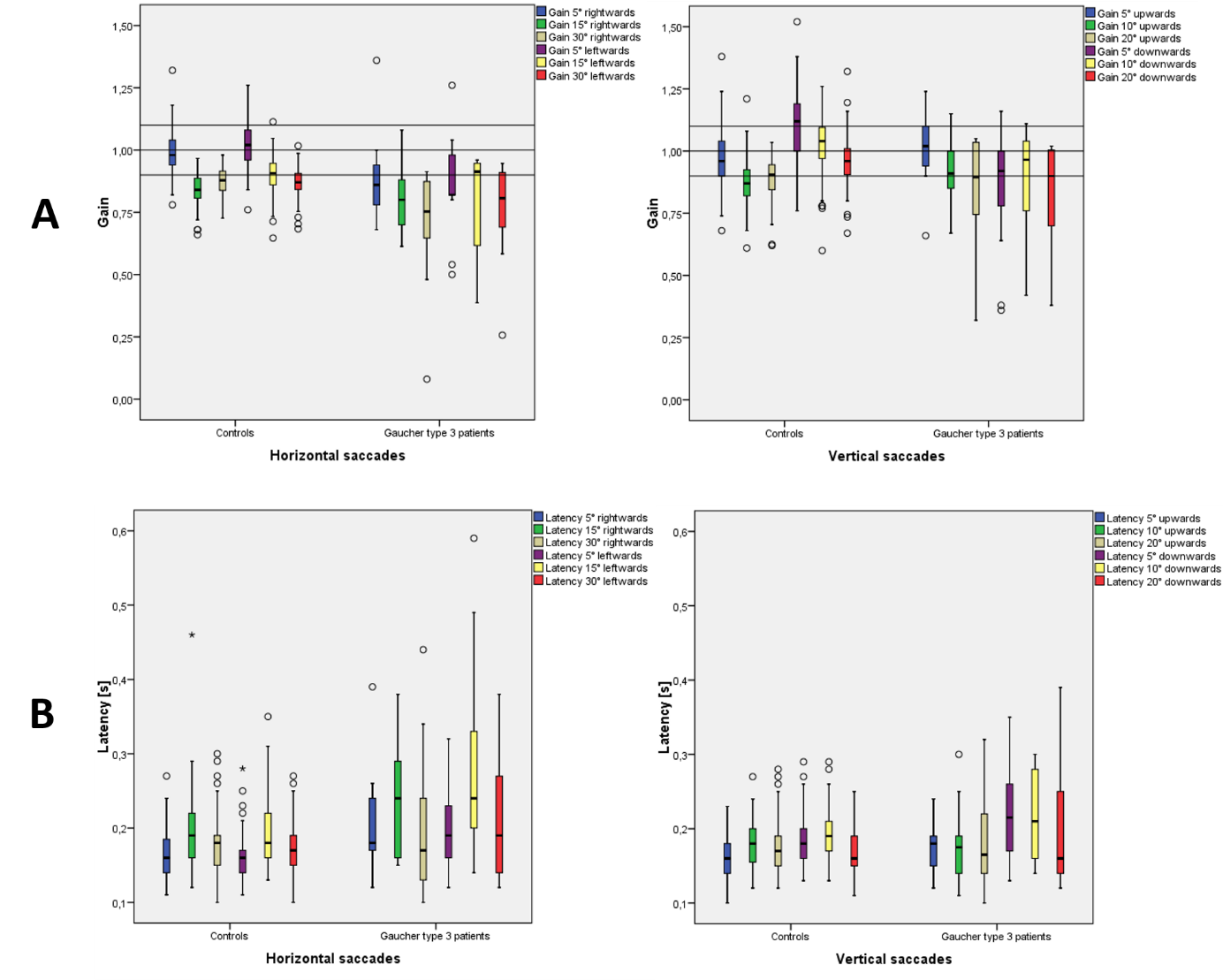
**

*Gain (A) and Latency (B) (y-axis) are displayed for Gaucher type 3 patients vs. the control group for different target eccentricities. The whiskers extend to the minimum and maximum, when there are no outliers. Outliers (and extreme values) are displayed as circles (asterisks), meaning values defined by a distance of more than 1.5 times (3 times) the interquartile distance from the box (from the median). The lower boundary of the box is the 25th percentile (25 % quartile), the line within the box indicates the 50th percentile (median) and the upper boundary represents the 75th percentile (75 % quartile). The arithmetic mean is shown as asterisk. In all age groups, the peak velocity increases with increasing target eccentricity.*
