# Supplementary material for: A comprehensive monocentric ophthalmic study with Gaucher disease type 3 patients: vitreoretinal lesions, retinal atrophy and characterization of abnormal saccades
Source: Orphanet J Rare Dis. 2019 Nov 14;14:257. doi: 10.1186/s13023-019-1244-9 (PMC6857165; doi:10.1186/s13023-019-1244-9)
Supplement: Supplementary file 3 — Additional file 3: Correlation analysis between saccade parameters and other neurologic items (Table). [file 13023_2019_1244_MOESM3_ESM.docx]

**Additional file 3: Correlation analysis between saccade parameters and other neurologic items.**

|  | *Peak velocity* | | *Gain* | | *Clinical evaluation* | |
| --- | --- | --- | --- | --- | --- | --- |
|  | *r* | *p* | *r* | *p* | *r* | *p* |
| *mSST* | [-0.33;-0.58] | 0.029 once | [-0.37;+0.71] | 0.004 once | +0.5 | 0.04 |
| *SARA* | [-0.19;-0.57] (h)  [-0-42;-0.79] (v) | 0.004 once  0.001-0.011 | [+0.02;+0.54] (h)  [-0.03;-0.75] (v) | 0.002-0.023 | +0.67 (h)  +0.86 (v) | 0.005  0.000 |
| *IQ* | [+0.63;+.81] (v) | 0.000-0.017 |  |  | [-0.7;-0.72] | 0.002-0.003 |
| *Disease duration* | [-0.57;-0.80] | 0.001-0.035 (v) | [-0.14;-0.72] | 0.004 once | [+0.26-+0.36] |  |
| *Clinical Evaluation* | [+0.78;+0.89] (v)  [+0.42;+0.7] (h) | 0.000-0.002 (v)  0.005-0.04 (h) | (-)  (-) | (-)  (-) | (-)  (-) | (-)  (-) |

*Data not shown were not coherent, or not applicable (-).*
