# Supplementary material for: A comprehensive monocentric ophthalmic study with Gaucher disease type 3 patients: vitreoretinal lesions, retinal atrophy and characterization of abnormal saccades
Source: Orphanet J Rare Dis. 2019 Nov 14;14:257. doi: 10.1186/s13023-019-1244-9 (PMC6857165; doi:10.1186/s13023-019-1244-9)
Supplement: Supplementary file 4 — Additional file 4: Neurologic items in Gaucher type 3 patients (Table). [file 13023_2019_1244_MOESM4_ESM.docx]

**Additional file 4: Neurologic items in Gaucher type 3 patients.**

| *Variables* | *Findings* |
| --- | --- |
| Total number of patients | 16 |
| Duration of disease | median 15 years (range 3.6 to 38.8 years) |
| Phenotye severity (genetic mutation)   - Mild/protective - Intermediate - Severe | 6 times (1x G202/D409H, 3 x L444P/D409H, 2 x others)  9 times (homozygous L444P)  1 time (unknown) |
| mSST  mSST (adjusted for the item gaze palsy) | median 2.25 (range 0.5 to 19.5) of 36 pts.  median 2.0 (range 0 to 18.5) of 35 pts. |
| SARA | median 1.5 (range 0 to 18, statistical outlier 39) of 40 pts. |
| IQ   - Slight mental retardation (IQ 50-69) - Learning disability (IQ 70-84) - Average intelligence (IQ 85-110) | 4 times  4 times  8 times |
| Medication | 4 patients with anticonvulsive medication |
